# Spatial distributions and determinants of intimate partner violence among married women in Ethiopia across administrative zones

**DOI:** 10.1371/journal.pone.0310039

**Published:** 2025-02-19

**Authors:** Meseret Tadesse Fetene, Senait Cherie Adgeh, Haile Mekonnen Fenta

**Affiliations:** 1 Department of Psychiatry, College of Medicine and Health Science, Bahir Dar University, Bahir Dar, Ethiopia; 2 Departments of Statistics, College of Science, Bahir Dar University, Bahir Dar, Ethiopia; The University of Texas Health Science Center at Houston, UNITED STATES OF AMERICA

## Abstract

**Background:**

Intimate partner violence (IPV) against women is highly prevalent in the world, especially in low-middle-income countries including Ethiopia. Studies so far assessing risk factors for IPV often use the classical model without geographical location information and spatial effects. This study aimed to estimate the overall prevalence and associated risk factors of intimate partner violence among Ethiopian administrative zones.

**Method:**

The 2016 Ethiopian Demographic and Health Survey (EDHS) data were used. The primary outcome of the study was the experience of different types of IPV: physical, emotional, and sexual by ever-married women aged 15–49 years. We adopted a generalized multilevel mixed model with IPV as an outcome variable and zones as random effects.

**Results:**

The prevalence of physical, emotional, and sexual violence in Ethiopia are respectively 20.38%, 22.31%, and 7.58%. The result indicated that 1,423 (30.15%) of respondents had experienced at least one of the three types of IPV. Women who had older age had more children, had lower decision-making power, and had a husband who was a drinker and had controlling behavior were more likely to experience any forms of IPV. Significant zone-wise spatial variations of IPV were also observed.

**Conclusions:**

The distribution of IPV in married women varies among Ethiopian administrative zones. Several factors were associated with IPV, therefore, interventions targeting the hotspot areas and specific determinant factors should be implemented by the concerned bodies to reduce IPV among married women in the population.

## Background

Intimate partner violence (IPV) is defined by the World Health Organization (WHO) as “any behavior within an intimate relationship that causes physical, emotional or sexual harm” [1]. Different literatures use the terms IPV: as gender-based violence, violence against women, women domestic abuse, domestic violence, and spousal violence interchangeably [2–4]. It is a fundamental violation of women’s human rights, as well as a significant public health crisis, and a major obstacle to sustainable millennium developmental goals [5]. Even though IPV exists in all societies, its prevalence varies widely by areas [6, 7]. Globally, almost one-third (30 to 38%) of all women who have been in a relationship have experienced physical, emotional or sexual violence and 38% of all murders of women are committed by intimate partners [6, 8]. But the report from the 2010 Global Burden of Disease, the largest prevalence of IPV is found in central sub-Saharan Africa, with 65.6% of women have experienced it [6, 9]. A meta-study conducted from international demographic health surveys (DHS) between 2010 and 2017 across 46 low and middle-income countries, including Ethiopia, among women aged 15–49 years reported varied prevalence across countries from 3.5% in Armenia and Comoros to 46% in Afghanistan [10, 11]. But studies in Ethiopia systematically reviewed, the lifetime prevalence of physical, sexual, and emotional violence ranged from 31 to 76.5%,19.2 to59%, and 51.7%, respectively [12].

Research in developed countries showed that women exposed to IPV are more likely to experience symptoms of depression, anxiety, psychogenic non-epileptic seizures, and psychotic disorders [13]. In a study done in South Africa among women, who had IPV, 45.3% reported clinically main symptoms of depression and 30.0% suicidal ideation [14]. Association between IPV and mental illness have been reported in different literatures [15–17]. Women experiencing physical violence, childhood sexual abuse, emotional violence, and spousal control were factors independently associated with depressive episodes [17]. Common risk factors for IPV include: low levels of education, exposure to child maltreatment, witnessing parental violence, antisocial personality disorder, substance abuse, multiple partners/infidelity, attitudes accepting of violence [8], gender inequality, and poverty [18], early marriage [19, 20], younger and not empowered women, and those living in rural areas are more vulnerable to IPV exposure in most countries [11]. The risk factors identified from a limited number of studies in Ethiopia include; older women, were married before the age of 18 years, witnessed inter-parental violence during their childhood, had a partner who drank alcohol, and living in a community with high IPV-accepting norms [21], living in rural areas, divorced, primary and secondary education, 25–39 years old, being poor are vulnerable to IPV [22]. Spouses have a positive attitude towards women’s autonomy, educated men and men who have higher access to information are less likely to perpetrate violence [23].

Due to its profound and long-lasting consequences for survivors and families, the international community has increasingly recognized the immediate attention to improve global policy action to avoid violence against women [24]. Despite the growing international attention, however, there is still limited investment in IPV research and coordination in measuring progress toward the 2030 SDGs in most LMICs [25]. More investments in research to build evidence based on the associations between IPV and its factors are essential for policymakers. Though several studies demonstrated that Ethiopia has recorded promising progress in reducing levels of IPV over the past decades, the challenges and achievements of different administrative Zones have not been studied yet. Hence detecting the problem of IPV and its variation among administrative Zones provides deeper insight into the country’s health priorities for IPV among women for zonal health departments to plan, follow up, monitor, and evaluate health activities at the lower level which helps policymakers to design focused intervention strategies. There are some cultural variations among administrative Zones, which result in different practices regarding IPV at the Zone level [26–32]. We therefore set out to determine the prevalence and risk factors for intimate partner violence and look for its distribution at different regions and administrative zones of Ethiopia using the most recent EDHS 2016 dataset.

## Methodology

### Study design

The data for this paper was drawn from the nationally representative cross-sectional study design, 2016 Ethiopian Demographic and Health Surveys (EDHS), described in detail at https://dhsprogram.com and we used the shapefiles of the second administrative zones of the country (https://africaopendata.org/dataset/ethiopia-shapefiles). In DHS, multistage sampling was used to select the sample for each survey: where the first step of the sampling procedure involved the selection of clusters (enumeration areas (EAs)), followed by systematic household sampling within the selected EAs. The number of clusters is the first stage which is selected from the list of enumeration areas (EAs) created in the recent population census of the country and the households that are randomly selected in each of EAs. From the selected households, women aged 15–49 years are selected for an in-depth interview [33]. In the first stage, 645 primary sampling units were chosen in the first stage (443 from rural areas and 202 from urban areas). In the second stage, an average of 28 households from each primary sampling unit were selected through systematic random sampling. The data focused on individual records from ever-married women aged 15 to 49, with 15,683 participants achieving a 95% response rate. For the domestic violence module, one married woman per household was interviewed, resulting in 5,860 women being selected and interviewed with a 97% response rate. The current study analyzed responses from a weighted sample of 4,720 ever-married women who completed the intimate partner violence questionnaire. Sampling weights were adjusted to account for the complex sampling procedures and ensure the results are nationally representative [34].

### Inclusion and exclusion criteria

This study was focused on the experience of intimate partner violence (IPV) among ever-married women in Ethiopia. It included all ever-married women aged 15–49 in the last five years preceding the surveys in the enumeration area who are currently married, divorced, or widowed. Exclusion criteria include missing values and respondents younger than 15 or older than 49.

**Fig 1:**
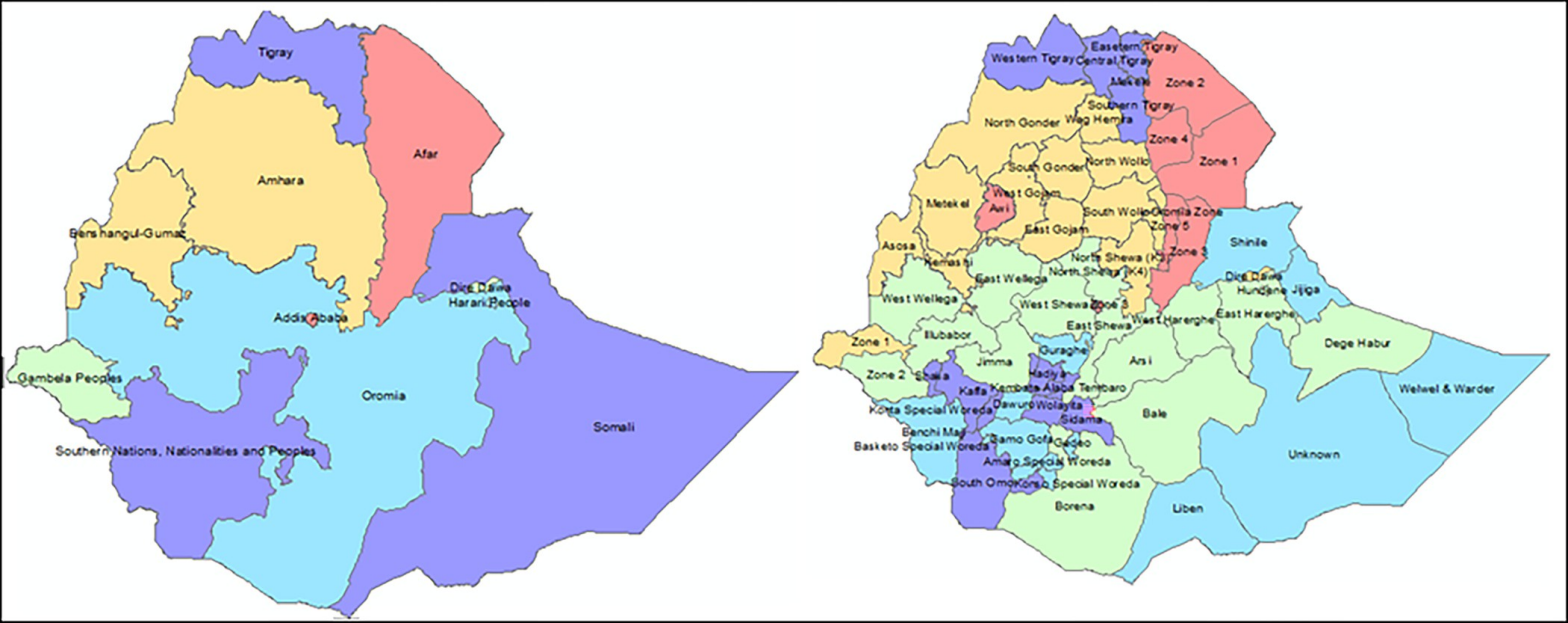
Map of Ethiopia and showing regions and zones. A) The eleven regions of Ethiopia; B) The 72 zones of Ethiopia.

Ethiopia is situated in the Horn of Africa from 30 to 140 and 330 to 480 E. The country is a low-income country in East Africa, is a completely landlocked country that has a surface area of 1.1 million km^2^ [35]. For administrative purposes, the country is divided into 11 regions; and a total of 72 administrative areas called zones, a setting for which the entire analysis is carried out (Fig 1).

### Variables

**Fig 2:**
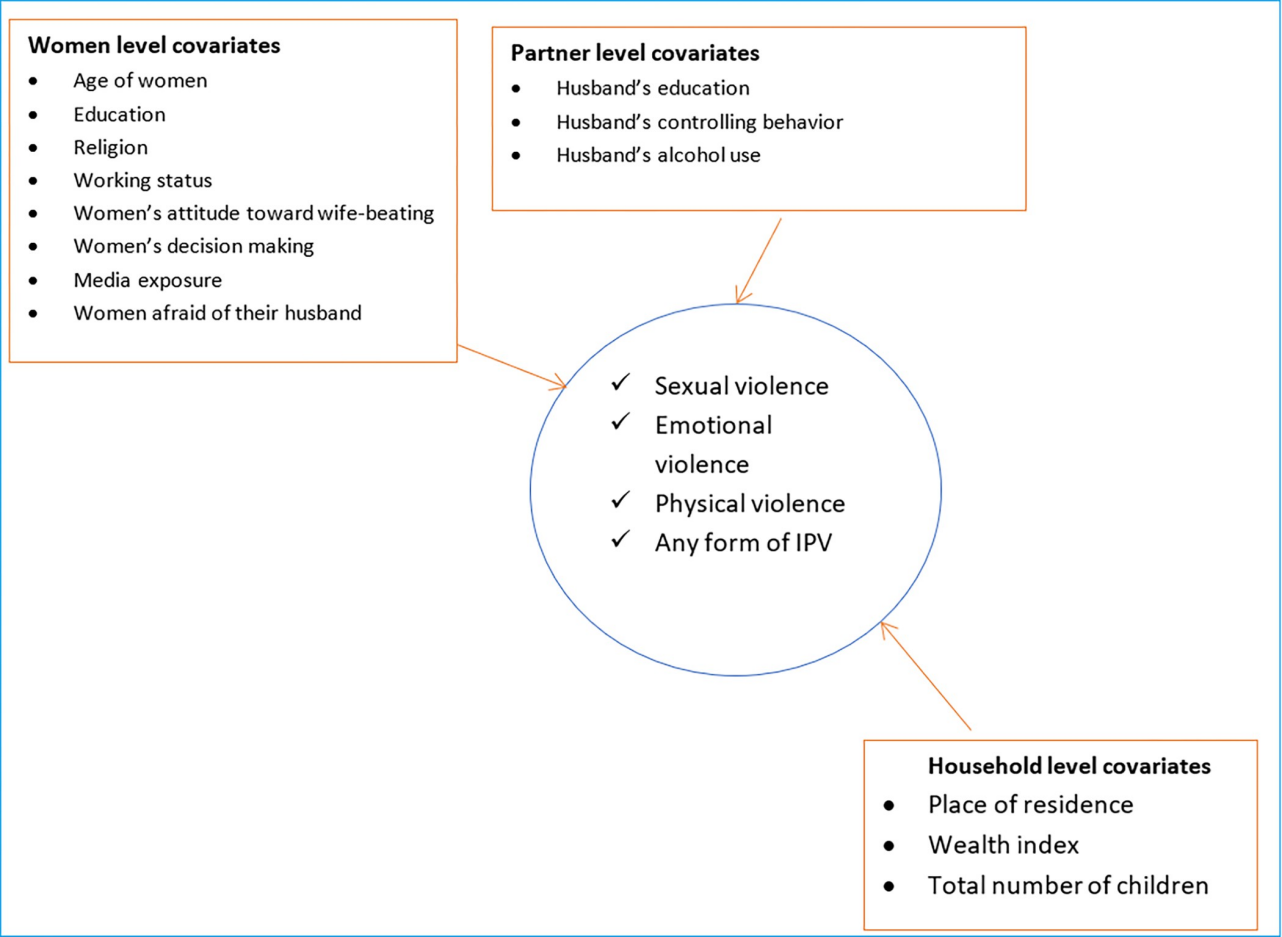
Conceptual framework for features description.

The outcome variable for this study includes single or multiple forms of physical, emotional, and sexual partner violence which was assessed using women’s self-reported responses to the questions depending on the modified Conflict of Tactic Scales of Status [36]. This information is crucial for understanding which women have experienced multiple types of violence in their lifetime. The emotional, physical, sexual, and any combinations of violence had a Cronbach’s alpha of 0.82, 0.67, 0.72; and, 0.68 respectively, indicating overall good test performance of the interview questions (Table 1). The IPV is defined as women who have experienced at least one event of physical, emotional, or sexual violence since the age of 15 years [25, 36, 37]. The covariates include all socio-demographic characteristics that were taken as the independent variables for the different survey years, which were selected from other kinds of literature [5–8, 10, 11, 16] (Fig 2).

**Table 1:** The tool used to measure IPV in the demographic and health surveys.

Question/item	IPV type
Push you, shake you, or throw something at you?	physical IPV
Slap you?	
Twist your arm or pull your hair	
Punch you with his/her first or with something that could hurt you?	
Kick you, drag you, or beat you up?	
Try to choke you or burn you on purpose?	
Threaten or attack you with a knife, gun or any other weapon?	
Physically force you to have sexual intercourse with him even when you did not want to?	Sexual IPV
Physically force you to perform any other sexual acts you did not want to?	
Force you with threats or in any other way to perform sexual acts you did not want to?	
Say or do something to humiliate you in front of others?	Emotional IPV
Threaten to hurt or harm you or someone close to you?	
Insult you or make you feel bad about yourself?	

### Spatial heterogeneity of IPV prevalence

First we computed and mapped the crude prevalence of IPV in the 72 administrative zones. The prevalence of IPV was estimated at each zone and we used ESRI Desktop 10.3 [38] to generate the maps of IPV prevalence using a kriging interpolation technique, a methodology widely used in spatial mapping [39–41].

### Statistical analysis

The data management was done using Stata version 16. The data were weighted to make them representative and to provide better statistical estimates. We adopted the generalized linear mixed model (GLMM) [42–46] to examine the effect of the women, husband, and household characteristics on IVP measures for married women 15–49 age in Ethiopia. The adopted GLMM model is:

$$g\left(\mu_{ij}\right)=logit(\mu_{ij})=\log\left(\frac{\mu_{ij}}{1-\mu_{ij}}\right)=\log(\frac{P(y_{ij}=1)}{P(y_{ij}=0)})=\eta_{ij},$$

where $\eta_{ij}=\beta_{0}+\beta_{1}x_{1ij}+\cdots +\beta_{k}x_{kij}+u_{0j},X_{1ij}$, through *X*_*kij*_, note the k explanatory variables measured on women, husbands and households. The *μ*_*ij*_ and 1-*μ*_*ij*_ are respectively the probability of a women experiencing IPV and not experiencing IVP (j = 1,…, 72 zones, i = 1,…, *n*_*j*_ women within each zone): where *β*_0_ is the log odds of intercept; *β*_1_ … *β*_*k*_ are effect sizes of women and household-level covariates *u*_0*j*_ are random errors at Zone level. The distribution of $u_{0j}∼N(0,\sigma_{u0}^{2})$. The intra-class correlation (ICC) was computed using between-Zone variance and within the Zone, variance (ICC = (σu2σu2+σe2) [47–49].

### Ethical consideration

This study used datasets of national representative demographic health surveys. Therefore, ethical is approval not required. But, datasets for this study were requested by providing a clear explanation about the objectives and necessity of this study. We registered and requested the DHS dataset to the online database (www.dhsprogram.com) and received an authorization letter to download the requested datasets.

## Results

**Fig 3:**
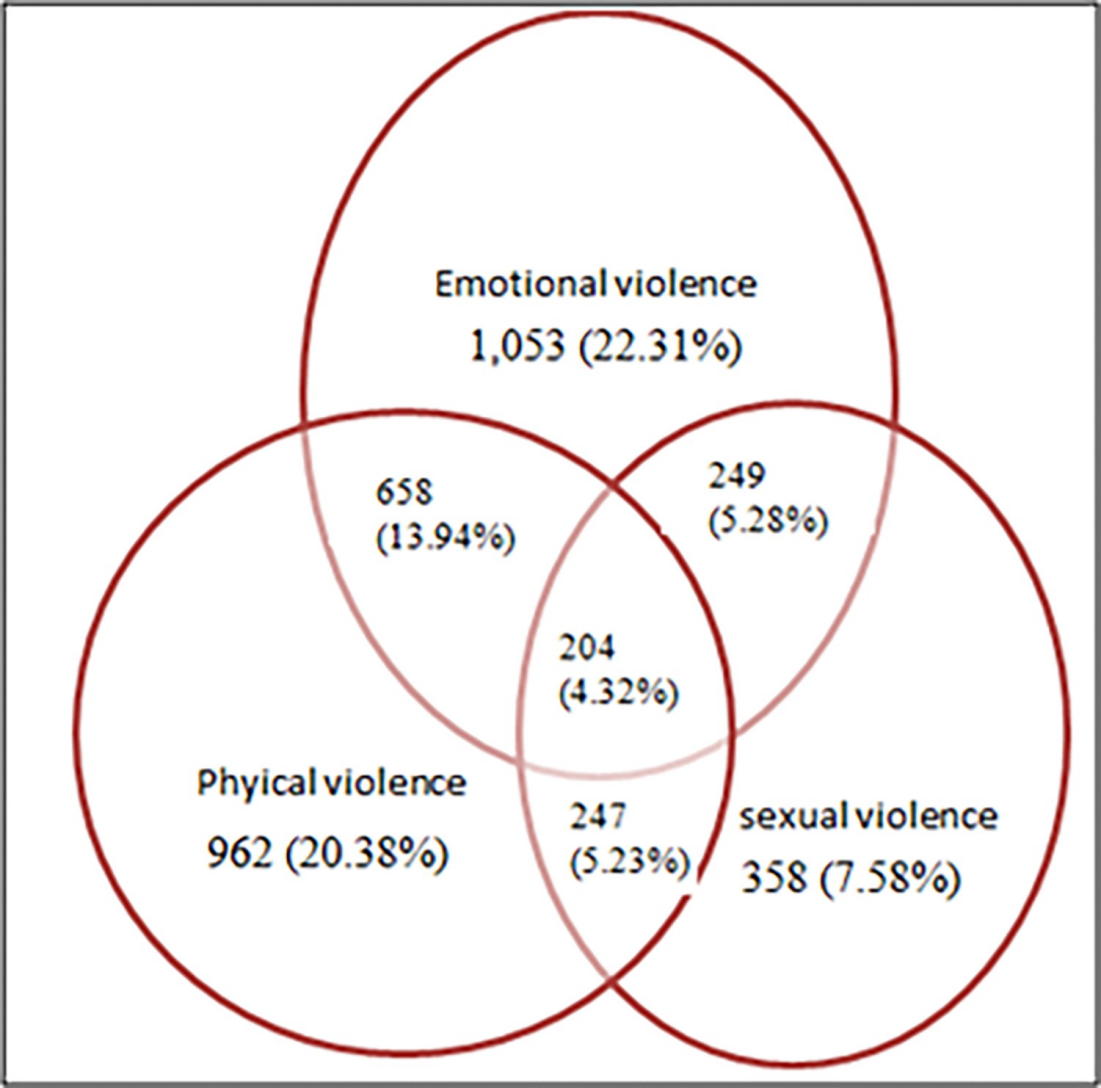
Overlap prevalence revealing frequencies and percentage of physical, emotional and sexual partner violence against married women in Ethiopia.

The result in Fig 3 revealed the experience of different forms of intimate partner violence, single or in combinations among women who reported experience of one or more forms of IPV. The result indicated that 1,053 (22.31%), 962 (20.38%) and 358 (7.58%) of women reported that they have experienced emotional, physical and sexual intimate partner violence alone respectively. However, 658 (13.94%) of the married women had experienced both physical and emotional violence and 204 (4.32%) of women who had experienced all forms of intimate partner violence. Besides, the result shows that 1,379 (30.35%) of women who had experienced any forms of intimate partner violence.

### Spatial results

**Fig 4:**
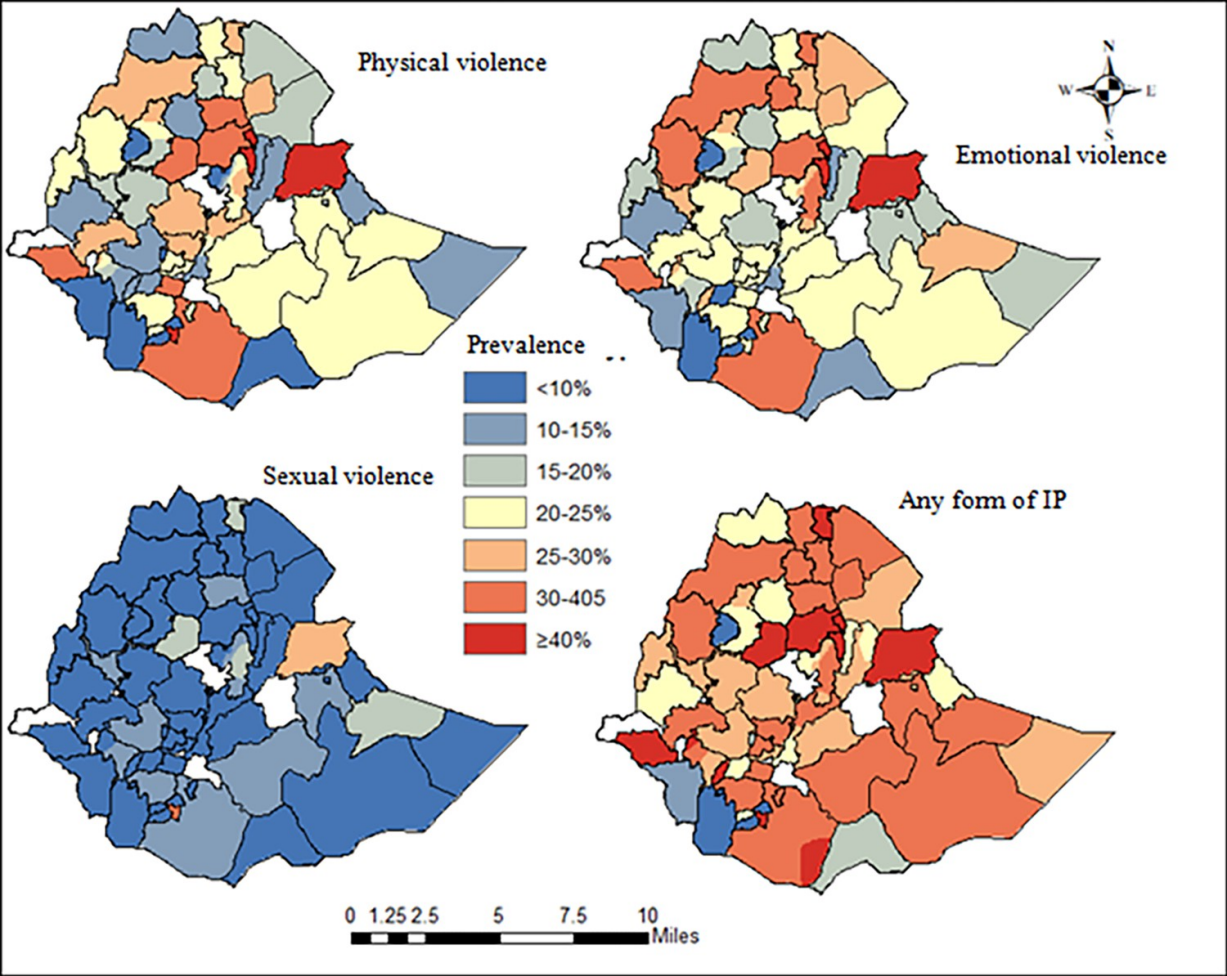
The prevalence of different types of IPV among the 72 administrative zones in Ethiopia.

The proportion of IPV varied a lot between the administrative zones in Ethiopia. Shinile in Somali region was the zone with most physical IPV, while women in North Shewa and most of the zones in SNNP regions (Amaro, Basketo, Konso and Yem special woredas) administrative zones reported lowest Physical IPV prevalence. Generally, there was a zonal variations in the proportion of physical, sexual, emotional, and any form of IPV among the administrative zones in Ethiopia (Fig 4).

The analysis included 4,720 eligible married women. The descriptive statistics was done to present the proportion of socio-demographic characteristics and women who had experienced any of the IPV for each category in the covariates. More than half of all married women had no formal education 2,735 (57.94) and 1,953 (47.37%) of their husbands had no education. Almost 37% (n = 1,716) of women were employed and almost 21% of the women were not empowered in household decision-making activities. Two thousand seventy-eight (44.03%) of the married women never afraid of their husbands. The prevalence of physical, sexual, and emotional violence among married women was 20.38%, 7.58%, and 22.31%, respectively. The least and most prevalent from IPV were sexual (7.58%) and emotional IPV (22.31%). One in every three (30.15%) married women experienced any form of IPV in their lifetime. Twenty percent, 8.37%, 23.33%, and 31% of women with no formal education respectively experienced physical, sexual, and emotional and any form of IPV and the prevalence decreased with increased values of education. Experience of any form of IPV was highest among the oldest age group, but lowest among married women aged 15–24 years. The prevalence of the sexual, physical, emotional, and any form of IPV was lower among women living in the urban areas, and those belong to the higher wealth quintiles. Married women with controlling behavior from their partner/spouse, women with no decision making participation, women without media exposure, husband’s alcohol use, and women afraid of their spouse, had a higher prevalence of all forms of IPV. A statistically strong relationship between decision-making in the household and experience of any form of IPV was demonstrated. Women who reported not involving in decision making had a higher odds of experiencing IPV compared to those who usually made decision. The highest prevalence of IPV (46.55%) was observed among women who had a husband who was a drinker compared to those who had no drinker husband (24.44%). Similarly, the IPV prevalence varies according to the husband’s education level. The highest IPV percentage among women was observed whose husband’s a primary education (33.18%) had compared with those who had no education (28.93%) and higher education (24.54%) (Table 2).

**Table 2:** The prevalence of partner violence by socio-demographic characteristics among ever-partnered or married 15–49 year old women in Ethiopia (n = 4720).

Variables	Sample, n (%)	Physical, n (%)	Sexual, n (%)	Emotional, n(%)	IPV, n (%)
Outcome variables		962 (20.38)	358 (7.58)	1,053 (22.31)	1,423 (30.15)
**Women level covariates**					
age in 5-year groups					
15–24	1,140 (24.15)	201(17.63)	67 (5.88)	208 (18.25)	295 (25.88)
25–34	2,012 (42.63)	419 (20.83)	158 (7.85)	434 (21.57)	610 (30.32)
35–44	1,219 (25.83)	263 (21.58)	102 (8.37)	305 (25.02)	391 (32.08)
45–49	349 (7.39)	79 (22.64)	31 (8.88)	106 (30.37)	127 (36.39)
Education					
No Education	2,735 (57.94)	556 (20.33)	229 (8.37)	638 (23.33)	815 (30.99)
Primary	1,320 (27.97)	288 (21.82)	100 (7.58)	304 (23.03)	399 (31.42)
Secondary	433 (9.17)	86 (19.86)	21 (4.85)	78 (18.01)	117 (27.79)
Higher	232 (4.92)	32 (13.79)	8 (3.45)	33 (14.22)	48 (21.52)
Religion					
Orthodox	1,805 (38.24)	391 (21.66)	166 (9.20)	437 (24.21)	589 (32.63)
Muslim	1,993 (42.22)	345 (17.31)	116 (5.82)	374 (18.77)	510 (25.59)
Protestant	835 (17.69)	197 (23.59)	69 (8.26)	210 (25.15)	285 (34.13)
Traditional And Others	87 (1.84)	29 (33.33)	7 (8.05)	32 (36.78)	39 (44.83)
Working status					
No	3,004 (63.64)	560 (18.64)	232 (7.72)	638 (21.24)	856 (28.50)
Yes	1,716 (36.36)	402 (23.43)	126 (7.34)	415 (24.18)	567 (33.04)
women’s attitude to wife-beating					
Does not justify wife-beating	136 (2.880	32 (23.53)	12 (8.82)	30 (22.06)	4 (33.09)
Justifies wife-beating	4,584 (97.12)	930 (20.29)	346 (7.55)	1,023 (22.32)	1,378 (30.06)
women decision making					
no participation	972 (20.59)	280 (28.81)	105 (10.80)	274 (28.19)	363 (37.35)
moderate participation	3,225 (68.33)	561 (17.40)	213 (6.60)	652 (20.22)	894 (27.72)
high level	523 (11.08)	121 (23.14)	40 (7.65)	127 (24.28)	166 (31.74)
media exposure					
no	2,865 (60.70)	588 (20.52)	246 (8.59)	663 (23.14)	878 (30.65)
yes	1,855 (39.30)	374 (20.16)	112 (6.04)	390 (21.02)	545 (29.38)
women afraid husband					
never afraid	2,078 (44.03)	215 (10.35)	76 (3.66)	229 (11.02)	348 (16.75)
most of the time	783 (16.59)	336 (42.91)	136 (17.37)	340 (43.44)	434 (55.43)
sometimes	1,859 (39.39)	411 (22.11)	146 (7.85)	484 (26.04)	641 (34.48)
**Partner level covariates**					
husband’s Education					
No Education	1,953 (47.37)	361 (18.48)	143 (7.32)	429 (21.97)	565 (28.93)
Primary	1,287 (31.22)	279 (21.68)	106 (8.24)	317 (24.63)	427 (33.18)
Secondary	493 (11.96)	86 (17.44)	27 (5.48)	88 (17.85)	121 (24.54)
Higher	390 (9.46)	51 (13.08)	11 (2.82)	50 (12.82)	77 (19.74)
Partner’s controlling behavior					
No controlling behavior	2,236 (47.37)	175 (7.83)	64 (2.86)	190 (8.5)	297 (13.28)
Has controlling behavior	2,484 (52.63)	787 (31.68)	294 (11.84)	863 (34.74)	1,126 (45.33)
husband alcohol use					
no	3,502 (74.19)	546 (15.59)	198 (5.65)	633 (18.08)	856 (24.44)
yes	1,218 (25.81)	416 (34.15)	160 (13.14)	420 (34.48)	567 (46.55)
**household level covariates**					
Place of residence					
Rural	3,509 (74.34)	731(20.83)	295 (8.41)	798 (22.74)	1,088 (31.01)
Urban	1,211 (25.66)	231 (19.08)	63 (5.20)	255 (21.06)	335 (27.66)
Wealth Index					
Poorest	1,412 (29.92)	261 (18.48)	109 (7.72)	292 (20.68)	400 (28.33)
Poorer	729 (15.44)	159 (21.81)	72 (9.88)	174 (24.14)	234 (32.10)
Middle	660 (13.98)	147 (22.27)	63 (9.55)	166 (25.15)	229 (34.70)
Richer	610 (12.92)	149 (24.43)	54 (8.85)	159 (26.07)	208 (34.10)
Richest	1,309 (27.73)	246 (18.79)	60 (4.58)	260 (19.86)	352 (26.89)
total number of children					
0	471 (9.98)	70 (14.86)	26 (5.52)	76 (14.14)	102 (21.66)
1–4	2,130 (45.13)	450 (21.13)	145 (6.81)	451 (21.17)	642 (30.14)
4 or more	2,119 (44.89)	442 (20.86)	187 (8.82)	526 (24.82)	679 (32.04)

Table 3 presents the results of the mixed effect logistic regression analysis with 95% level of confidence, which shows the association of fixed effects and the random effects (zones) for ever experience of physical, sexual, and emotional IPV among married women aged 15–49 years. We found a significant association between the ages of married women with emotional and any form of IPV. Compared to married women aged 15–24 years, women aged 35–44 60% (AOR = 1.60; 95% CI 1.17, 2.19) and 45% (AOR = 1.45; 95% CI 1.09, 1.64) were more likely to experience emotional and at least one form of IPV respectively. The educational status of women had an impact on emotional violence only. Women with higher education are 40% less likely to experience emotional violence compared with no formal education. Women with decision-making autonomy in the household were 27% (AOR 0.73, 95% CI: 0.55, 0.98) and 25% (AOR 0.75: 95% CI: 0.58, 0.97) less likely to report experiencing physical and emotional violence respectively. More importantly, compared to women who were never afraid of their husbands, women who were most of the time afraid of their husbands were more likely to report experiencing physical, sexual, and emotional partner violence. Particularly, women who afraid of their husband sometimes and most of the time were about 3 times (AOR 2.85, 95% CI: 2.37, 3.43) and 5 times (AOR 4.94, 95% CI: 3.94, 6.20) more likely to report experiencing of IPV respectively. Women married to a partner who drank alcohol, were about 4 times (AOR 3.58, 95% CI: 2.82, 4.55), 3 times (AOR 1.81, 3.48), and 3 times (AOR 2.57, 95% CI: 2.06, 3.22) more likely to report experiencing physical, sexual and emotional intimate partner violence respectively (Table 3).

**Table 3:** Factors associated with experiencing physical, emotional, sexual and intimate partner violence using GLMM.

Variables	Physical	Sexual	Emotional	Intimate partner (IPV)
**women level covariates**	AORs 95% CI	AORs 95% CI	AORs 95% CI	AORs 95% CI
age in 5-year groups				
15–24	1	1	1	1
25–34	1.04 (0.80, 1.36)	1.14 (0.75, 1.73)	1.10 (0.84, 1.41)	1.13 (0.89, 1.42)
35–44	1.32 (0.96, 1.83)	1.30 (0.80, 2.14)	1.60 (1.17, 2.19)***	1.45 (1.09, 1.64)**
45–49	0.99 (0.64, 1.54)	1.25 (0.66, 2.37)	1.72 (1.15, 2.58)**	1.44 (0.99, 2.11)
Education				
No Education			1	1
Primary			1.02 (0.83, 1.26)	1.18 (0.95, 1.46)
Secondary			0.77 (0.55, 1.09)	1.44 (0.99, 1.08)
Higher			0.60 (0.39, 0.62)**	1.12 (0.67, 1.87)
Religion				
Orthodox	1		1	1
Muslim	1.62 (1.24, 2.12)***		1.16 (0.91, 1.49)	1.32 (1.05, 1.66)*
Protestant	1.52 (1.15, 2.01)***		1.22 (0.94, 1.60)	1.40 (1.09, 1.79)**
Traditional And Others	2.31 (1.29, 4.15)***		2.20 (1.25, 3.88)**	2.40 (1.38, 4.15)**
Respondent Currently Working				
No	1	1	1	1
Yes	1.28 (1.05, 1.55)**	0.81 (0.60, 1.08)	1.13 (0.93, 1.36)	1.17 (0.98, 1.39)
women decision making				
No	1		1	
Yes	0.73 (0.55, 0.98)*		0.75 (0.58, 0.97)**	
media exposure				
no			1	
yes			0.92 (0.74, 1.15)	
women afraid husband				
never afraid	1	1	1	1
most of the time	4.97 (3.87, 6.39)***	4.23 (2.93, 6.10)***	4.65 (3.65, 5.93)***	4.94 (3.94, 6.20)***
sometimes	2.40 (1.93, 2.98)***	2.32 (1.65, 3.26)***	2.90 (2.36, 3.57)***	2.85 (2.37, 3.43)***
**Partner level covariates**				
husband’s education				
No Education	1	1	1	1
Primary	1.03 (0.82, 1.28)	1.11 (0.81, 1.51)	1.03 (0.83, 1.26)	1.03 (0.85, 1.25)
Secondary	0.91 (0.64, 1.29)	1.21 (0.72, 2.04)	0.77 (0.55, 1.09)	0.75 (0.55, 1.03)
Higher	0.79 (0.51, 1.24)	0.70 (0.33, 1.52)	0.600 (0.39, 0.92)**	0.67 (0.45, 0.98)*
Partner’s controlling behavior (MC)				
No controlling behavior	1	1	1	1
Has controlling behavior	4.06 (3.32, 4.97)***	3.38 (2.47, 4.64)***	4.78 (3.93, 5.79)***	4.43 (3.75, 5.25)***
husband alcohol use				
no	1	1	1	
yes	3.58 (2.82, 4.55)***	2.51 (1.81, 3.48)***	2.57 (2.06, 3.22***	3.30 (2.66, 4.09)***
**household level covariates**				
Place of residence				
Rural			1	
Urban			0.75 (0.52, 1.08)	
Wealth Index				
Poorest	1	1		1
Poorer	1.03 (0.78, 1.35)	1.12 (0.78, 1.61)		0.94 (0.74, 1.20)
Middle	1.03 (0.77, 1.37)	1.03 (0.70, 1.52)		1.08 (0.84, 1.39)
Richer	0.94 (0.96, 1.27)	0.76 (0.49, 1.18)		0.78 (0.60, 1.03)
Richest	0.95 (0.62, 1.45)	0.45 (0.23, 0.89)		0.77 (0.53, 1.12)
total number of children				
0	1		1	1
1–3	1.35 (0.91, 2.01)		1.21 (0.83, 1.76)	1.32 (0.94, 1.84)
4 and more	1.25 (0.81, 1.96)		1.24 (0.81, 1.91)	1.28 (0.87, 1.88)
Random Effects components				
ICC (95% CI)	0.17 (0.13, 0.22)***	0.24 (0.18, 0.33)***	0.13 (0.09, 0.17)***	0.16 (0.13, 0.20)***

GLMM: generalized linear mixed effect model, aOR = adjusted odds ratio, CI = confidence interval

^c^P < 0.a0001

^b^P < 0.001

^a^P < 0.05

The best linear unbiased predictor (BLUP) shows that there are variations of IPV (physical, emotional, and sexual) among the administrative zones of Ethiopia. Based on the BLUP for the zone-level random effect, the zones were ranked and the best five (those with lowest standardized BLUP values) and top 5 “worst” (those with the highest standardized BLUP values) performing zones in terms of IPV were identified. Hence, women who live in Metekel zone in Benshangul gumz region, Guraghe zone in SNNP, Dege Habur zone in Somali region, South wollo and North Gondar in Amhara region had a higher IPV. However, women who lived in Western Tigray, Jimma and East shewa in Oromia region, Jigjiga in Somali region, and Zone 3 in Addis Ababa had the lowest IPV (Table 4).

**Table 4:** Model based comparisons of different intimate partner violence among administrative zones in Ethiopia in married women.

		Physical		Emotional		Sexual		IPV	
Regions of Ethiopia	Districts/ zones	BLUP	Ranking	BLUP	Ranking	BLUP	Ranking	BLUP	Ranking
Addis Ababa	AAUnknown	0.0883	64	0.0068	48	0.0910	64	0.1951	64
	AAZone 1	0.0435	54	0.0106	49	0.0436	53	0.1068	54
	AAZone 2	-0.0958	9	-0.0350	11	-0.0937	8	-0.2155	7
	AAZone 3	-0.1137	6	-0.0435	3	-0.1124	5	-0.2606	5
	AAZone 4	0.0107	47	0.0237	63	0.0062	44	0.0496	51
	AAZone 5	0.0019	44	-0.0050	31	0.0063	45	0.0123	41
	AAZone 6	-0.0730	13	-0.0416	4	-0.0697	13	-0.1753	11
Afar	Zone 1	-0.0603	17	0.0207	61	-0.0527	18	-0.0833	20
Afar	Zone 2	**-0.1202**	**4**	0.0476	67	-0.1238	3	-0.1874	8
Afar	Zone 3	-0.0962	8	-0.0076	28	-0.0917	9	-0.1866	9
Afar	Zone 4	0.0759	59	0.0129	52	0.0745	58	0.1723	59
Afar	Zone 5	-0.0147	34	-0.0095	25	-0.0142	33	-0.0294	33
Amhara	Awi	-0.0379	23	-0.0155	22	-0.0373	24	-0.0816	21
	Bar Dar Sp. Zone	0.0157	49	0.0029	44	0.0154	48	0.0430	49
	East Gojam	0.1355	67	0.0179	59	0.1329	66	0.2952	67
	North Gonder	0.1663	68	0.0814	70	0.1546	67	0.4113	69
	North Shewa (K3)	-0.0137	35	0.0287	65	-0.0133	34	0.0107	40
	North Wollo	0.2085	71	0.0023	43	0.2048	70	0.4246	70
	Oromia Zone	-0.0100	36	-0.0037	34	-0.0098	36	-0.0145	34
	South Gonder	-0.0764	12	-0.0177	19	-0.0713	12	-0.1564	14
	South Wollo	0.1811	70	0.0537	68	0.1816	69	0.4254	71
	Wag Hemira	0.0201	51	0.0234	62	0.0209	50	0.0734	52
	West Gojam	-0.0632	16	-0.0396	6	-0.0643	15	-0.1581	13
Benshangul-Gumaz	Asosa	-0.0150	33	-0.0621	1	-0.0190	31	-0.0871	19
	Kemashi	-0.0023	40	0.0174	58	-0.0030	40	0.0211	44
	Metekel	0.0640	57	0.1115	72	0.0549	54	0.2393	66
Dire Dawa	Dire Dawa	-0.0007	42	-0.0272	14	-0.0120	35	-0.0308	32
Gambela Peoples	GZone 1	0.0916	65	0.0276	64	0.0898	63	0.2180	65
Gambela Peoples	GZone 2	-0.0394	22	-0.0055	30	-0.0396	23	-0.0756	24
Harari People	Hundene	-0.0727	14	-0.0157	21	-0.0654	14	-0.1449	16
Oromia	Arsi	-0.0342	25	-0.0086	26	-0.0295	27	-0.0633	27
	Bale	-0.0049	38	-0.0171	20	0.0006	42	-0.0123	35
	Borena	0.0174	50	-0.0045	33	0.0188	49	0.0407	48
	East Harerghe	0.0345	52	-0.0332	13	0.0386	52	0.0489	50
	East Shewa	-0.1145	5	-0.0562	2	-0.1134	4	-0.2751	3
	East Wellega	-0.0039	39	0.0140	53	-0.0024	41	0.0166	43
	Illubabor	0.0803	62	0.0050	45	0.0788	60	0.1731	60
	Jimma	-0.1485	2	-0.0208	17	-0.1422	2	-0.3025	2
	North Shewa (K4)	-0.0912	10	0.0114	50	-0.0875	10	-0.1582	12
	West Harerghe	-0.0637	15	-0.0390	9	-0.0596	16	-0.1533	15
	West Shewa	0.0599	56	-0.0347	12	0.0553	55	0.0894	53
	West Wellega	-0.0245	29	-0.0203	18	-0.0214	30	-0.0572	29
Somali	Dege Habur	0.0403	53	0.0603	69	0.0330	51	0.1426	55
	Jijiga	-0.1248	3	-0.0411	5	-0.1065	6	-0.2634	4
	Liben	-0.1058	7	-0.0393	7	-0.1039	7	-0.2401	6
	Shinile	0.0652	58	0.0061	46	0.0648	57	0.1452	57
	Unknown	0.1103	66	-0.0378	10	0.1109	65	0.1924	63
	Welwel & Warder	-0.0486	19	-0.0046	32	-0.0530	17	-0.0971	18
SNNP	Amaro Special Woreda	-0.0158	32	0.0063	47	0.124	72	-0.0005	38
	Basketo Special Woreda	-0.0011	41	-0.0033	37	-0.0093	38	-0.0047	37
	Benchi Maji	-0.0331	26	-0.0112	24	-0.0311	25	-0.0665	26
	Burji Special Woreda	-0.0536	18	-0.0392	8	-0.0514	19	-0.1352	17
	Dawuro	-0.0818	11	-0.0216	16	-0.0837	11	-0.1782	10
	Derashe Special Woreda	-0.0006	43	-0.0013	39	0.0036	43	0.0107	39
	Gamo Gofa	0.0596	55	0.0192	60	0.0563	56	0.1442	56
	Gedeo	-0.0177	31	0.0173	57	-0.0142	32	-0.0056	36
	Guraghe	0.3133	72	0.1087	71	0.3045	71	0.7354	72
	Hadiya	0.0790	60	0.0145	55	0.0816	61	0.1840	62
	Kaffa	-0.0452	20	-0.0036	36	-0.0402	22	-0.0800	22
	Kembata Alaba Tembaro	0.0801	61	0.0147	56	0.0782	59	0.1820	61
	Konso Special Woreda	0.0134	48	-0.0021	38	-0.0068	39	0.0135	42
	Konta Special Woreda	0.0069	45	0.0120	51	0.0077	46	0.0356	46
	Shaka	0.0093	46	-0.0037	35	0.0083	47	0.0229	45
	Sidama	0.0851	63	-0.0070	29	0.0841	62	0.1713	58
	South Omo	-0.0244	30	-0.0231	15	-0.0247	29	-0.0632	28
	Wolayita	0.1672	69	-0.0078	27	0.1589	68	0.3273	68
	Yem Special Woreda	-0.0290	28	0.0005	42	-0.0281	28	-0.0476	30
Tigray	Central Tigray	-0.0373	24	-0.0006	41	-0.0405	21	-0.0694	25
	Easetern Tigray	-0.0326	27	0.0143	54	-0.0301	26	-0.0394	31
	Mekele	-0.0414	21	-0.0009	40	-0.0438	20	-0.0772	23
	Southern Tigray	-0.0052	37	0.0421	66	-0.0094	37	0.0365	47
	Western Tigray	-0.1514	1	-0.0112	23	-0.1536	1	-0.3072	1

## Discussion

This study aimed to examine the prevalence of intimate partner violence among Ethiopian administrative zones and identify the determinant factors using data from the 2016 EDHS and shapefiles. Women in Ethiopia and around the globe experience different forms of violence (such as physical, emotional, and sexual) throughout their lives. However, a single intimate violence index such as sexual, emotional, or physical does not show the holistic picture of violations among married women. To alleviate this problem, we adopted a multifaceted single index known as intimate partner violence. Using this index, we investigated the disparities of Ethiopian married women’s violation status in space which shows the administrative zones, the second level of the Ethiopian administrative level where the social service delivery decision-making process is made.

Most of the studies conducted in Ethiopia had only reported geographical variations of IPV at higher (country/region) aggregated levels [17, 19, 21, 22, 50], and zonal level variation is rarely examined. A closer look into the contents of the studies shows that their IPV data is masked in higher-level geographical aggregates, and hurt lower levels (zones in this context). This is inconsistent with the decentralized system of governance in Ethiopia. The zone is the administrative level where operation planning, resource allocation, and implementation of health services (including women empowerment for decision-making) are made. Hence identifying the problem of IPV and its variation among administrative zones would provide deeper insight into the country’s health priorities of women in the population. Particularly, this would help Zonal health departments to make informed decisions and actions in their planning, follow-up, monitoring, and evaluation of women’s empowerment and decision-making ability at lower levels.

We found that IPV is a considerable public health problem and the prevalence was 30.15 indicating that one in every three women had experienced at least one form of IPV. The overall prevalence was higher than studies conducted in Saudi Arabia and Benin [51–53], but lower than studies conducted in sub-Saharan Africa, Zimbabwe, Gambia, and studies in 46 low-middle income countries (LMICs) [11, 54–56]. The presence of all spousal violence varied considerably across the administrative zones of the country. At the women level covariates, older age, no formal education level, no media experience, working status and afraid of their partner were positively associated with some forms of IPV. Women of higher age were more likely to experience emotional and IPV. However, other researchers found that the risk of experiencing IPV was higher among the lower age groups [57–59]. The possible reasons for the contradictory findings could be cultural and area level differences between the study samples and IPV. Women married to a spouse who drank alcohol had increased the odds of physical, sexual, emotional and IPV [57, 58, 60]. This is due to the strong impact of unlimited alcohol intake which may change the behavior of husbands. Partner’s controlling behavior in a relationship was found to be a protective factor against physical, sexual, emotional and IPV.

Moreover, the spatial heterogeneity and inequality of IPV was analyzed and mapped at the second administrative levels in Ethiopia. The presence of more than 80 ethnic groups in Ethiopia’s 72 zones means that cultural practices and gender norms differ. The zones with the highest IPV status and the highest degree of inequality were identified. It is already known that IPV prevalence differs among geographic areas. Many studies limitation have been conducted in Ethiopia, but most of them were focused in only regions or states. There is clearly a variation of IPV at the second administrative zones even within the same regions, hence, we focused on the second level administrative areas (zones).

The study has both strengths and limitations. This large dataset made it possible to apply the high-level identify the important factors. However, this study has some limitations. Firstly, we considered only one recent DHS dataset, and hence we did not model the variables over time. Secondly, the data is cross-sectional so we can only make conclusions on statistical association (not causality). Thirdly, the research could be susceptible to social desirability and potential recall bias due to its reliance on self-reported data.

## Conclusions

This study aimed to investigate the magnitude of intimate partner violence and its influencing factors by utilizing national EDHS data, as part of the crucial efforts to accomplish Sustainable Development Goals (SDGs) related to gender equality and women’s empowerment, ultimately contributing to ending violence against women. The spatial units for intimate partner violence analysis is “zones” (n = 72 zones in Ethiopia). The result showed that the proportion of women who had experienced physical, sexual, emotional or at least one form of this IPV was high in Ethiopia. Our findings suggest there are zone-wise variations in the prevalence of IPV. Taking into account the context and cultural norms among zones in Ethiopia, the IPV cases could even be under-reported. Our finding shows where and towards which populations IPV resources should be allocated could help national health policymakers develop appropriate sets of interventions or prevent the use of incorrect interventions for intimate partner violence control and prevention in Ethiopia. Being younger age, women’s decision-making autonomy, never being afraid of their husbands, no husband’s alcohol drink, partners without controlling behavior were negatively associated with any form of IPV. Hence improving the power of women’s decision-making autonomy, Precision public health approaches are important for targeting health policies to zones most affected by IPV. Moreover, the best-worst performing zones were identified and this study recommends further investigation into these zones which didn’t show any progress in improving intimate partner violence among married women in Ethiopia.

## Abbreviations

EDHS: Ethiopian Demographic and Health Survey
SNNP: South Nation Nationalities and People
AOR: Adjusted Odds Ratio

## References

[1] García-Moreno C., et al., WHO multi-country study on women’s health and domestic violence against women. 2005: World Health Organization.

[2] Baldasare A., Gender-based violence: Focus on Africa. SAI-From Vision to Results, 2012.

[3] Ahmad A. and Jaleel A., Prevalence and correlates of violence against women in Nepal: findings from Nepal Demographic Health Survey, 2011. Advances in Applied Sociology, 2015. 5(04): p. 119.

[4] Hindin M.J., Kishor S., and Ansara D.L., Intimate partner violence among couples in 10 DHS countries: Predictors and health outcomes. 2008: Macro International Incorporated.

[5] Garcia-Moreno C., et al., Prevalence of intimate partner violence: findings from the WHO multi-country study on women’s health and domestic violence. The lancet, 2006. 368(9543): p. 1260–1269. 10.1016/S0140-6736(06)69523-8 17027732

[6] Organization, W.H., Global and regional estimates of violence against women: prevalence and health effects of intimate partner violence and non-partner sexual violence. 2013: World Health Organization.

[7] Sulak T.N., Saxon T.F., and Fearon D., Applying the theory of reasoned action to domestic violence reporting behavior: the role of sex and victimization. Journal of Family Violence, 2014. 29(2): p. 165–173.

[8] Organization, W.H., Violence against women: Intimate partner and sexual violence against women: Intimate partner and sexual violence have serious short-and long-term physical, mental and sexual and reproductive health problems for survivors: Fact sheet. 2014, World Health Organization.

[9] Ellsberg M., et al., Intimate partner violence and women’s physical and mental health in the WHO multi-country study on women’s health and domestic violence: an observational study. The lancet, 2008. 371(9619): p. 1165–1172. 10.1016/S0140-6736(08)60522-X 18395577

[10] Andersson N., et al., Risk factors for domestic physical violence: national cross-sectional household surveys in eight southern African countries. BMC Women’s Health, 2007. 7(1): p. 11. doi: 10.1186/1472-6874-7-11 17631689 PMC2042491

[11] Coll C.V., et al., Intimate partner violence in 46 low-income and middle-income countries: an appraisal of the most vulnerable groups of women using national health surveys. BMJ global health, 2020. 5(1): p. e002208. doi: 10.1136/bmjgh-2019-002208 32133178 PMC7042580

[12] Semahegn A. and Mengistie B., Domestic violence against women and associated factors in Ethiopia; systematic review. Reproductive health, 2015. 12(1): p. 78. 26319026 10.1186/s12978-015-0072-1 PMC4553009

[13] Meekers D., Pallin S.C., and Hutchinson P., Intimate partner violence and mental health in Bolivia. BMC women’s health, 2013. 13(1): p. 28. 23799992 10.1186/1472-6874-13-28 PMC3698003

[14] Gibbs A., Dunkle K., and Jewkes R., Emotional and economic intimate partner violence as key drivers of depression and suicidal ideation: A cross-sectional study among young women in informal settlements in South Africa. PloS one, 2018. 13(4): p. e0194885. doi: 10.1371/journal.pone.0194885 29659595 PMC5901771

[15] Neter J., et al., Applied linear statistical models. 1996.

[16] Campbell J.C., Health consequences of intimate partner violence. The lancet, 2002. 359(9314): p. 1331–1336. doi: 10.1016/S0140-6736(02)08336-8 11965295

[17] Deyessa Kabeta N., Intimate partner violence and depression among women in rural Ethiopia. 2010, Umeå University. 10.1186/1745-0179-5-8 PMC2689215 19397834

[18] Karamagi C.A., et al., Intimate partner violence against women in eastern Uganda: implications for HIV prevention. BMC public health, 2006. 6(1): p. 284. doi: 10.1186/1471-2458-6-284 17116252 PMC1660563

[19] Erulkar A., Early marriage, marital relations and intimate partner violence in Ethiopia. International perspectives on sexual and reproductive health, 2013: p. 6–13. doi: 10.1363/3900613 23584463

[20] Speizer I.S. and Pearson E., Association between early marriage and intimate partner violence in India: a focus on youth from Bihar and Rajasthan. Journal of interpersonal violence, 2011. 26(10): p. 1963–1981. doi: 10.1177/0886260510372947 20587462 PMC3741349

[21] Tiruye T.Y., et al., Determinants of intimate partner violence against women in Ethiopia: A multi-level analysis. PLoS one, 2020. 15(4): p. e0232217. doi: 10.1371/journal.pone.0232217 32330193 PMC7182270

[22] Chernet A.G. and Cherie K.T., Prevalence of intimate partner violence against women and associated factors in Ethiopia. BMC women’s health, 2020. 20(1): p. 22. doi: 10.1186/s12905-020-0892-1 32028961 PMC7006182

[23] Lawoko S., Predictors of attitudes toward intimate partner violence: A comparative study of men in Zambia and Kenya. Journal of Interpersonal violence, 2008. 23(8): p. 1056–1074. doi: 10.1177/0886260507313972 18292405

[24] García-Moreno C., et al., Addressing violence against women: a call to action. The Lancet, 2015. 385(9978): p. 1685–1695. doi: 10.1016/S0140-6736(14)61830-4 25467579

[25] Temmerman M., Research priorities to address violence against women and girls. The Lancet, 2015. 385(9978): p. e38–e40. doi: 10.1016/S0140-6736(14)61840-7 25467581

[26] Gebreyesus S.H., et al., Local spatial clustering of stunting and wasting among children under the age of 5 years: implications for intervention strategies. Public health nutrition, 2016. 19(8): p. 1417–1427. doi: 10.1017/S1368980015003377 26700548 PMC10270919

[27] Collaborators G.R.F., Global, regional, and national comparative risk assessment of 79 behavioural, environmental and occupational, and metabolic risks or clusters of risks, 1990–2015: a systematic analysis for the Global Burden of Disease Study 2015. Lancet (London, England), 2016. 388(10053): p. 1659. doi: 10.1016/S0140-6736(16)31679-8 27733284 PMC5388856

[28] Corsi D.J., et al., Shared environments: a multilevel analysis of community context and child nutritional status in Bangladesh. Public health nutrition, 2011. 14(6): p. 951–959. doi: 10.1017/S1368980010003356 21310102

[29] Griffiths P., et al., A tale of two continents: a multilevel comparison of the determinants of child nutritional status from selected African and Indian regions. Health & place, 2004. 10(2): p. 183–199. doi: 10.1016/j.healthplace.2003.07.001 15019912

[30] Fenta H.M., Zewotir T., and Muluneh E.K., Disparities in childhood composite index of anthropometric failure prevalence and determinants across Ethiopian administrative zones. PloS one, 2021. 16(9): p. e0256726. doi: 10.1371/journal.pone.0256726 34555038 PMC8459952

[31] Fetene M.T., Fenta H.M., and Tesfaw L.M., Spatial heterogeneities in acute lower respiratory infections prevalence and determinants across Ethiopian administrative zones. Journal of Big Data, 2022. 9(1): p. 1–16.

[32] Fenta H.M., Zewotir T., and Muluneh E.K., Spatial regression models to assess variations of composite index for anthropometric failure across the administrative zones in Ethiopia. Plos one, 2024. 19(2): p. e0282463. doi: 10.1371/journal.pone.0282463 38416735 PMC10901317

[33] Aliaga A. and Ren R., The optimal sample sizes for two-stage cluster sampling in demographic and health surveys. 2006: ORC Macro.

[34] (CSA), C.S.A., Demographic and Health Survey 2016.

[35] Milkias P., Ethiopia. 2011: Abc-Clio.

[36] Straus M.A. and Douglas E.M., A short form of the Revised Conflict Tactics Scales, and typologies for severity and mutuality. Violence and victims, 2004. 19(5): p. 507–520. doi: 10.1891/vivi.19.5.507.63686 15844722

[37] Chernet A.G. and Cherie K.T., Prevalence of intimate partner violence against women and associated factors in Ethiopia. BMC women’s health, 2020. 20(1): p. 1–7. 32028961 10.1186/s12905-020-0892-1 PMC7006182

[38] Kennedy M.D., Introducing geographic information systems with ARCGIS: a workbook approach to learning GIS. 2013: John Wiley & Sons.

[39] Berke O., Exploratory disease mapping: kriging the spatial risk function from regional count data. International Journal of Health Geographics, 2004. 3(1): p. 1–11. 15333131 10.1186/1476-072X-3-18 PMC516784

[40] Goovaerts P., Geostatistical analysis of disease data: estimation of cancer mortality risk from empirical frequencies using Poisson kriging. International Journal of Health Geographics, 2005. 4(1): p. 1–33. 16354294 10.1186/1476-072X-4-31 PMC1360096

[41] Carrat F. and Valleron A.-J., Epidemiologic mapping using the “kriging” method: application to an influenza-like epidemic in France. American journal of epidemiology, 1992. 135(11): p. 1293–1300. 1626546 10.1093/oxfordjournals.aje.a116236

[42] McCulloch C.E., Searle S.R., and Neuhaus J.M., Generalized, linear, and mixed models. Hoboken. NJ: Wiley. QA, 2008. 279: p. M38.

[43] Skrondal A. and Rabe‐Hesketh S., Prediction in multilevel generalized linear models. Journal of the Royal Statistical Society: Series A (Statistics in Society), 2009. 172(3): p. 659–687.

[44] Teshale A.B., et al., Anemia and its associated factors among women of reproductive age in eastern Africa: A multilevel mixed-effects generalized linear model. Plos one, 2020. 15(9): p. e0238957. doi: 10.1371/journal.pone.0238957 32915880 PMC7485848

[45] Neuhaus J.M., Kalbfleisch J.D., and Hauck W.W., A comparison of cluster-specific and population-averaged approaches for analyzing correlated binary data. International Statistical Review/Revue Internationale de Statistique, 1991: p. 25–35.

[46] Breslow N.E. and Clayton D.G., Approximate inference in generalized linear mixed models. Journal of the American statistical Association, 1993. 88(421): p. 9–25.

[47] Goldstein H., Multilevel statistical models. Vol. 922. 2011: John Wiley & Sons.

[48] Hox J.J., Moerbeek M., and Van de Schoot R., Multilevel analysis: Techniques and applications. 2017: Routledge.

[49] Wu L., Mixed effects models for complex data. 2009: CRC Press.

[50] Semahegn A. and Mengistie B., Domestic violence against women and associated factors in Ethiopia; systematic review. Reproductive health, 2015. 12: p. 1–12. 26319026 10.1186/s12978-015-0072-1 PMC4553009

[51] Alzahrani T.A., Abaalkhail B.A., and Ramadan I.K., Prevalence of intimate partner violence and its associated risk factors among Saudi female patients attending the primary healthcare centers in Western Saudi Arabia. Saudi medical journal, 2016. 37(1): p. 96. doi: 10.15537/smj.2016.1.13135 26739983 PMC4724688

[52] Kpozehouen A., et al., Perception of Beninese on intimate partner violence: evidence from 2011–2012 Benin demographic health survey. BMC women’s health, 2018. 18: p. 1–9. 30115038 10.1186/s12905-018-0633-x PMC6097337

[53] Mossie T.B., et al., Mapping the disparities in intimate partner violence prevalence and determinants across Sub-Saharan Africa. Frontiers in public health, 2023. 11: p. 1188718. doi: 10.3389/fpubh.2023.1188718 37448663 PMC10337829

[54] Ahinkorah B.O., Dickson K.S., and Seidu A.-A., Women decision-making capacity and intimate partner violence among women in sub-Saharan Africa. Archives of Public Health, 2018. 76: p. 1–10. 29423218 10.1186/s13690-018-0253-9 PMC5787915

[55] Jabbi A., et al., Prevalence and factors associated with intimate partner violence against women in The Gambia: a population-based analysis. Women & health, 2020. 60(8): p. 912–928. doi: 10.1080/03630242.2020.1767264 32419660

[56] Iman’ishimwe Mukamana J., Machakanja P., and Adjei N.K., Trends in prevalence and correlates of intimate partner violence against women in Zimbabwe, 2005–2015. BMC international health and human rights, 2020. 20: p. 1–11. 31959182 10.1186/s12914-019-0220-8 PMC6971918

[57] Kishor S. and Johnson K., Profiling domestic violence: A multi-country study. 2004: MEASURE DHS+, ORC Macro.

[58] Abramsky T., et al., Findings from the SASA! Study: a cluster randomized controlled trial to assess the impact of a community mobilization intervention to prevent violence against women and reduce HIV risk in Kampala, Uganda. BMC medicine, 2014. 12(1): p. 1–17. doi: 10.1186/s12916-014-0122-5 25248996 PMC4243194

[59] Fahmy H.H. and Abd El-Rahman S.I., Determinants and health consequences of domestic violence among women in reproductive age at zagazig district, egypt. J Egypt Public Health Assoc, 2008. 83(1–2): p. 87–106. 18992205

[60] Yusuf O., et al., Physical violence among intimate partners in Nigeria: A multi level analysis. Journal of Public Health and Epidemiology, 2011. 3(5): p. 240–247.

