# Supplementary material for: Spatial distributions and determinants of intimate partner violence among married women in Ethiopia across administrative zones
Source: PLoS One. 2025 Feb 19;20(2):e0310039. doi: 10.1371/journal.pone.0310039 (PMC11838895; doi:10.1371/journal.pone.0310039)
Supplement: Attachment — Submitted filename: Response to Reviewers.docx [file pone.0310039.s001.docx]

**RESPONSES TO THE EDITORS’ AND REVIEWERS’ COMMENT**

**Date: 07/17/2024**

**Manuscript title:** Spatial Distributions and determinants of intimate partner violence among married women in Ethiopia across administrative zones with manuscript ID: **PONE-D-23-29229**

### Dear Editor-in-Chief of Spatial Statistics,

We thank you and reviewers for your constructive comments and suggestions, which will significantly improve the quality and clarity of our manuscript. We have modified the manuscript according to the comments and suggestions made by the editor(s) and reviewers. Our detailed point-by-point response (in blue) to the comments raised by both the editor(s) and the reviewers is given below.

All the suggestions and corrections given by the editor (s) and reviewers are duly incorporated in the revised manuscript. The revisions are marked in coloured text in the manuscript file (Manuscript2). Specifically, the blue text indicates changes made in response to suggestions and comments of reviewer 1, the blue text indicates changes made according to the suggestions and comments of reviewer 2, and the red text indicates changes made according to the suggestions and comments of both. We still welcome comments and suggestions that may improve the quality of our manuscript.

We believe that our manuscript has been considerably improved and hope that our revised manuscript “**Spatial Distributions and determinants of intimate partner violence among married women in Ethiopia across administrative zones”** is suitable for publication in Plos one journal.

Thank you once again for your consideration of our work and for inviting us to submit the revised manuscript.

**Points raised by the academic editor**

1. Please ensure that your manuscript meets PLOS ONE's style requirements, including those for file naming.

Response: Thank you for bringing our attention to these resources. Our manuscript and associated files have been updated to reflect the style requirements specified in these documents.

2. Note from Emily Chenette, Editor in Chief of PLOS ONE, and Iain Hrynaszkiewicz, Director of Open Research Solutions at PLOS: Did you know that depositing data in a repository is associated with up to a 25% citation advantage (<https://doi.org/10.1371/journal.pone.0230416>)? If you’ve not already done so, consider depositing your raw data in a repository to ensure your work is read, appreciated and cited by the largest possible audience. You’ll also earn an Accessible Data icon on your published paper if you deposit your data in any participating repository (<https://plos.org/open-science/open-data/#accessible-data>).

Response: the data can be downloaded from the DHS website <https://dhsprogram.com>.

3. We noticed you have some minor occurrence of overlapping text with the following previous publication(s), which needs to be addressed:

- <https://doi.org/10.1371/journal.pone.0256726>

- <http://dx.doi.org/10.1136/bmjgh-2019-002208>

In your revision ensure you cite all your sources (including your own works), and quote or rephrase any duplicated text outside the methods section. Further consideration is dependent on these concerns being addressed.

Response: done

4. Thank you for stating the following in the Competing Interests section:

“No”

Please confirm that this does not alter your adherence to all PLOS ONE policies on sharing data and materials, by including the following statement: "This does not alter our adherence to  PLOS ONE policies on sharing data and materials.” (as detailed online in our guide for authors <http://journals.plos.org/plosone/s/competing-interests>).  If there are restrictions on sharing of data and/or materials, please state these. Please note that we cannot proceed with consideration of your article until this information has been declared.

Please include your updated Competing Interests statement in your cover letter; we will change the online submission form on your behalf.

Response: The amended Competing Interests Statement explicitly stating the details of the commercial funder has been included within the cover letter.

5. Please provide a complete Data Availability Statement in the submission form, ensuring you include all necessary access information or a reason for why you are unable to make your data freely accessible. If your research concerns only data provided within your submission, please write "All data are in the manuscript and/or supporting information files" as your Data Availability Statement.

Response: Thank you, our respected editor for your assistance and support. In short, the present study used data from the Ethiopian Demographic and Health Survey (EDHS) which is available from the DHS program website after the study's aim and objective were communicated. According to DHS dataset terms of use do not permit us to distribute this data as per data access instruction “The data must not be passed on to other researchers without the written consent of DHS.” For this reason, we include the following information under the “Data Availability” statement.

6. Your ethics statement should only appear in the Methods section of your manuscript. If your ethics statement is written in any section besides the Methods, please move it to the Methods section and delete it from any other section. Please ensure that your ethics statement is included in your manuscript, as the ethics statement entered into the online submission form will not be published alongside your manuscript.

Response: Thank you for your comment. As per your wise advice we moved the ethics statement in to the Method and Material section of the revised manuscript. Please see the revised manuscript

7. We note that Figures 1 and 3 in your submission contain [map/satellite] images which may be copyrighted. All PLOS content is published under the Creative Commons Attribution License (CC BY 4.0), which means that the manuscript, images, and Supporting Information files will be freely available online, and any third party is permitted to access, download, copy, distribute, and use these materials in any way, even commercially, with proper attribution. For these reasons, we cannot publish previously copyrighted maps or satellite images created using proprietary data, such as Google software (Google Maps, Street View, and Earth). For more information, see our copyright guidelines: <http://journals.plos.org/plosone/s/licenses-and-copyright>.

We require you to either (1) present written permission from the copyright holder to publish these figures specifically under the CC BY 4.0 license, or (2) remove the figures from your submission:

Response: Thank you, our respected editor, for your comment and wise advice. The figures (Fig 1 and 3) included in the present are not copyrighted or previously copyrighted maps or satellite images. Yet all Figures included in this study were our study findings/results showing the spatial distribution of IPV in Ethiopia across administrative zones and the third party is permitted to access, download, copy, and distribute (CC BY 4.0). In brief, the dataset we used to analyze spatial data was obtained from the publicly available DHS program ([www.dhsprogram.com](http://www.dhsprogram.com)) website, after the study objective was communicated and it can be published under the Creative Commons Attribution License (CC BY 4.0).

8. Please review your reference list to ensure that it is complete and correct. If you have cited papers that have been retracted, please include the rationale for doing so in the manuscript text, or remove these references and replace them with relevant current references. Any changes to the reference list should be mentioned in the rebuttal letter that accompanies your revised manuscript. If you need to cite a retracted article, indicate the article’s retracted status in the References list and also include a citation and full reference for the retraction notice.

Response: done

**In response to comments from Reviewer #1:**

Dear reviewer, thank you very much for taking the time to review our manuscript. We seriously considered your comments and suggestions and incorporated them into the revised manuscript. We gave due attention to the typographical errors, results, discussion sections, conclusions, and recommendations so that the manuscript has significantly improved.

Introduction:

1. It is necessary to not only mention that in Ethiopia, the identified prevalence of partner violence varies widely, with physical prevalence ranging from 31% to 76.5%, sexual prevalence from 19.2% to 59%, and emotional prevalence at 51.7%. It is important to specify the social norms and cultural characteristics that are related to this variation and high prevalence. Some of the articles cited by you mention religion, early marriage, and early initiation of sexual relationships as factors, but they are not described in the introduction.

Our response: Thank you for your valuable comment. The main aim of this study is to see the variations of IPV across the administrative zones (where there are cultural and social differences) which is stated (page no. 4, line no 13-18) in the introduction section. Moreover, religion was considered in the analysis and it has a significant impact on the IPV (page 9, Table 3).

2. A better justification of the research gap is required, to analyzing the data according administrative zones. It is unclear whether these administrative zones encompass different norms, cultures, languages, ethnicities, religions, or socioeconomic levels. It is necessary to describe the actual organization of the population and how there are related to the administrative zones and evaluate whether the analysis should be conducted based on other criteria. If necessary, include this information in the discussion and/or limitations.

Our response: Thank you for your comment. We improved the discussion section based on your comment and suggestions (Page 14 , line no. 3- 13).

3. Specify the study design. Is it a secondary analytical cross-sectional study based on the 2016 Ethiopia Demographic and Health Survey (DHS)?

Our response: Yes, it is, we specified it on page 4, line 21.

4. Include general characteristics of the 2016 Ethiopian DHS, such as the complex design being random, two-stage, and balanced. What was the primary sampling unit, the household?

Our response: thank you, done (page 4, line 22-31 and page 5: line no. 1-13).

5. There is no information in the manuscript about the population selection criteria for the study (inclusion and exclusion criteria). For example, were women included who were selected for the violence module and were married or cohabiting in the past 12 months? Were women excluded if they did not have privacy when answering violence-related questions? Or if they did not respond to questions about physical, sexual, or emotional violence? Include a flowchart showing the population selection criteria.

Our response: this is a very good point. We included in the methodology section (page 5, line no. 1-13)

6. Statistical power: It is important to describe the statistical power calculation to know if the information on factors associated with intimate partner violence according to administrative areas can include a type II error. It has only been analyzed up to regions, not administrative zones, in other of the articles that you cited that were also secondary analyzes of DHS Ethiopia
   Our response: thank you. This is applied research and we focused on the administrative zones, instead of regions. We justified the reasons in the previous response. The ICC was computed and added in the revised form of this manuscript with justification (Table 4).
7. Specify whether the different types of partner violence were assessed based on the question about violence by the partner in life or in the past 12 months?. Provide more precise operationalization of outcome variables.

Our response: thank you for your comment. It is in life time, which was mentioned in the result section, but we added it in the methodology section now (page, 5, line no. 21-23).

8. The independent variables considered in the analysis should be listed based on different studies that are not specified.

Our response: thank you for your suggestions, we added it as a conceptual framework (Fig.2).

Statistical analysis

9. The level of confidence considered in the statistical analysis is not specified (95%? 99%?)

Our response: it is 95%, it is mentioned in the footnote of Table 4 (p-value <0.05).

10. It is not specified whether any statistical test, such as the chi-square test with Rao-Scott correction, was used for the bivariate analysis in Table 2. Include the appropriate statistical test if applicable.

Our response: Table 2 is all about the descriptive statistics, which is the distribution of IPV across the covariates.

11. It is not specified what criteria were used to include the adjustment variables in Table 3. Was it based on statistical criteria? If so, specify it. For some forms of violence, different variables were adjusted (e.g., education level for emotional violence, but not for sexual or physical violence). It should be explained in the statistical analysis section whether this was due to multicollinearity or correlation analysis, how it was evaluated, and the criteria used for exclusion.

Our response: we used crude odds ratio in the context of GLMM and candidate variables for the given IPV form were included in the adjusted odds ratio multivariable cases. We added a sentence in the statistical analysis section now.

12. Specify the variables used to adjust the comparison model for different types of IPV in the administrative zones.
    Our response: it is different for different IPV forms, but all variables are included in Table 4 and the empty values in the table reveal that the variable is used to adjust the other variables (Table 4).
    Results.
    • Tables.
13. It is important to mention in the table footer of Table 3 what type of model was used to calculate those models (GLMM?). It would also be helpful to evaluate whether presenting both crude and adjusted models is feasible.

Our response: thank you, done.

14. For Table 4, justify whether the power is adequate for comparing zones without a type II error. It may be appropriate to perform an analysis at the regional level. Include in the table footer the specifications used to calculate the analysis, and explain the initials used (BLUP Ranking).

Our response: we computed the ICC in the model and it is statistically significant hence including zones as a random effect is meaningful.

Discussion.

15. It is important to contextualize the findings and discuss whether the geographic delimitation of administrative zones truly reflects the characteristics of partner relationships. Do the 80 ethnic groups in Ethiopia align with this delimitation?

Our response: this is a very important point of view. We used the DHS dataset and the shapefile of the zones, we tool the covariates from the DHS dataset and the random component (zone) from the shapefile. Neither the shapefile nor the DHS dataset consist the 80 ethnic group information. However, we improved the discussion section now as per your comment.

16. Does partner violence differ within these administrative zones when considering the social determinants model?1 It is possible that some subsets of these zones share common characteristics (social norms, cultural aspects, population structure, religion, socioeconomic level, etc.) and should be analyzed together and contextualized. Additionally, there may be differences within some of these zones.

Our response: thank you so much, this is another insight. The multilevel spatial models can be used to see the within and between variabilities. But in this paper, we focused on the multilevel aspect and we didn’t see the spatial modeling.

1Heise L, Greene ME, Opper N, Stavropoulou M, Harper C, Nascimento M, et al. Gender inequality and restrictive gender norms: framing the challenges to health. Lancet. 2019;393(10189):2440-54.

17. It is important to include the study limitations. Some potential limitations could be that the study is based on a cross-sectional design, which only allows for the identification of associations. Additionally, the violence data is self-reported, which may introduce reporting bias due to desirability bias, with individuals more likely to report severe or recent violence,
    What happen if there was another person when they asked about intimate partner violence? If there was no privacy, it was recorded in the survey; if so, were they excluded from the analysis?

Our response: thank you done

18. At Ethiopy DHS the question about violence it is reported who perpetrated the violence. Is it possible that the violence was committed by a former partner and not by his current partner? If there is that possibility, include it in limitations.

Our response: this is a very critical issue. But we considered lifetime violence and a woman may have one or more violence by their current or previous partner. This is because the questionnaire in the model asks about lifetime violence.

19. Are there any other self-reported variables that may be influenced by desirability bias?

Our response:

**In response to comments from Reviewer #2:**

1. Reviewer #2: Please check the formatting of the manuscript including spacing, punctuation, and grammar.
   Our response: thank you, we corrected it as per your suggestions.

Methods:

2. The section should clearly mention that the analysis adjusts for the complex sampling design of the EDHS data by applying sampling weights. It is important to reiterate that all estimates presented are weighted to account for the sampling design

Our response: thank correction has been made in the revised form of the manuscript.

3. The selection and justification of covariates included in the model are not adequately detailed.

Our response: we added the conceptual framework and justified it from different literature.

Results:

4. The interpretation of the spatial distribution results could be more detailed linking them to underlying factors (for example, cultural, socio-economic, and regional factors).

Our response: we treated the covariates as fixed effects and the zones as random components. We see the prevalence distribution of different forms of IPV across the zones, hence our objective was not to see the spatial effects of covariates on IPV across zones. This is a good research gap for further analysis.

Discussion:

5. While the discussion references existing literature, it could benefit from a more systematic comparison of the study’s findings with those of previous studies in detail. Currently the discussion is brief, but it could be strengthened by a more in-depth analysis of the findings, a clear connection to the Ethiopian context, and should delve into the reasons behind these findings comparing with the previous literature. Furthermore, the strengths and limitations of the study are not clearly outlined.

Our response: done
